# Antibodies and Vaccines against Botulinum Toxins: Available Measures and Novel Approaches

**DOI:** 10.3390/toxins11090528

**Published:** 2019-09-12

**Authors:** Christine Rasetti-Escargueil, Michel R. Popoff

**Affiliations:** Institut Pasteur, Département de Microbiologie, Unité des Toxines Bactériennes, 25 Rue du Docteur Roux, 75015 Paris, France; michel-robert.popoff@pasteur.fr

**Keywords:** botulinum neurotoxins (BoNTs), antitoxin, antibodies, vaccines, BoNT variants

## Abstract

Botulinum neurotoxin (BoNT) is produced by the anaerobic, Gram-positive bacterium *Clostridium botulinum*. As one of the most poisonous toxins known and a potential bioterrosism agent, BoNT is characterized by a complex mode of action comprising: internalization, translocation and proteolytic cleavage of a substrate, which inhibits synaptic exocytotic transmitter release at neuro-muscular nerve endings leading to peripheral neuroparalysis of the skeletal and autonomic nervous systems. There are seven major serologically distinct toxinotypes (A–G) of BoNT which act on different substrates. Human botulism is generally caused by BoNT/A, B and E. Due to its extreme lethality and potential use as biological weapon, botulism remains a global public health concern. Vaccination against BoNT, although an effective strategy, remains undesirable due to the growing expectation around therapeutic use of BoNTs in various pathological conditions. This review focuses on the current approaches for botulism control by immunotherapy, highlighting the future challenges while the molecular underpinnings among subtypes variants and BoNT sequences found in non-clostridial species remain to be elucidated.

## 1. Introduction

Botulinum neurotoxins (BoNTs) are among the most poisonous substances that exist, being part of the “dirty dozen” agents listed as possible bioweapons. BoNTs are mainly produced by bacteria of the genus *Clostridium* that are Gram-positive, anaerobic spore-forming microorganisms including *Clostridium botulinum*, atypical strains of other *Clostridium spp.,* such as *Clostridium butyricum and Clostridium baratii* [1]. The most common forms of botulism consist of foodborne botulism by oral intoxination, botulism by intestinal colonization (infant botulism and adult intestinal botulism) and wound botulism. Rare but often severe, naturally-occurring foodborne intoxications are still encountered worldwide as well as infant botulism cases due to intestinal colonization by *Clostridium* spores. Iatrogenic botulism cases are mainly due to the increased cosmetic usage of counterfeit and poorly calibrated BoNT. In rare cases, inhalational botulism may result from accidental release of aerosolized BoNT. Inhalational botulism was initially reported in laboratory workers and more recently in illicit drug users after intranasal use of cocaine as well as wound botulism after drug injection. All of these cause the same clinical syndrome of symmetrical cranial nerve palsies followed by descending, symmetric, flaccid paralysis of voluntary muscles as well as inhibition of secretions, which may progress to respiratory compromise and death. The chief clinical manifestation of botulism is a flaccid peripheral paralysis that can be fatal in the absence of intensive care unit support. Treatment is mainly symptomatic including meticulous intensive care with mechanical ventilation in the severe cases. Anti-BoNT antibodies are the only specific treatment which is effective if administered early after the onset of symptoms [2,3]. Despite numerous efforts, no small synthetic molecule as BoNT inhibitor has been approved for therapeutic use against botulism.

BoNTs are divided into more than seven toxinotypes (classically A to G and further recently identified toxinotypes) that are defined by specific neutralization with corresponding antibodies. BoNT/A is the deadliest biological substance currently known, with lethal dose values of 1 ng/kg in humans by the intravenous and subcutaneous routes and 3 ng/kg by the pulmonary route, according to experiments with non-human primates and investigations on naturally acquired botulism outbreaks [4]. BoNT/E-related intoxications are scarcer than those related to BoNT/A and BoNT/B, but the median LD50 of BoNT/E is estimated to be as low as that of BoNT/A, equal to 1.1 ng/kg in mice and monkeys by intraperitoneal route [5]. Like a few other non-proteolytic BoNT/B and BoNT/F toxinotypes, BoNT/E is secreted as a unique inactive chain by group II *C. botulinum* strains that requires activation by host proteases. This process—called nicking—is associated with a 100-fold increase in toxicity [6]. BoNT has been classified as a category A biothreat agent (by the United States Center of Disease Control and Prevention due to this extreme toxicity and ease of production [7]. The Soviet Union and Iraq have weaponized BoNTs and attempted splicing the BoNT gene into other bacteria as reported by U.N. Officers [8]. Furthermore, the risk of contamination of the food chain by BoNTs has been highlighted in several potential scenarios [9]. However, BoNT is used as a therapeutic agent for a growing number of indications including movement disorders, hemifacial spasm, essential tremor, tics, writer’s cramp, cervical dystonia, cerebral palsy, vascular cerebral stroke and more recently for chronic pain, migraine headache and overactive bladder.

This review focuses on the available anti-BoNTs antibodies and efforts made towards next generation vaccine against botulism, including DNA- and protein-based vaccines. Challenges posed in the future developments will be presented with a particular emphasis on projects focused on the development of recombinant antibodies to neutralize the most lethal types of BoNTs such as the AntiBotABE project.

## 2. Diversity and Structure of BoNTs

Most human botulism cases are caused by BoNT/A, B and E and to a lower extent BoNT/F [10,11]. Botulism due to BoNT/F is very rare, but most often associated with infant botulism, which is of importance for antibodies development [12]. A potential eighth novel BoNT, was reported as toxinotype H in 2014 [13], the designation of this novel toxin as a new serotype has been questioned due to its hybrid-like structure with regions of similarity to toxinotypes A and F and the fact that it is neutralized with toxinotype A antitoxin. It is now recognized as BoNT/FA or HA [13,14,15]. In addition, BoNT/like sequences have been identified in non-clostridial species such as *Weissella oryzae*, *Enterococcus faecium* and *Chryseobacterium piperi* [16] as well as a new BoNT serotype, tentatively named BoNT/X found in the *Clostridium Botulinum* strain 111 [17]. BoNT/Wo from *W. oryzae* adds to the BoNT diversity as a recognized novel toxinotype that cleaves VAMP2 similarly to BoNT/B, D, F and G [17,18,19]). The neurotoxin gene cluster recently identified within *E. faecium* encodes for a novel putative eBoNT/J (also called BoNT/En) but more importantly, the public health implications and potential therapeutic use of the novel BoNT types remain to be defined [20]. To add to this complexity, sequence analysis has allowed to distinguish numerous variants within each BoNT toxinotypes (more than 40) named “subtypes” (BoNT/A1,/A2, BoNT/B1,/B2 etc.) [1,20].

BoNTs are produced as large protein complexes combining a neurotoxic subunit with a non-toxic non-hemagglutinin (NTNH) component, and with either hemagglutinin (HA) components or OrfX proteins. Despite their sequence complexity, BoNTs share a similar structure consisting of a light chain (LC, 50 kDa) and a heavy chain (H, 100 kDa) linked by a disulfide bond. The crystal structures of BoNT/A, /B and /E show a tri-modular architecture with each domain fulfilling a “chaperone-like” role for the other domains [21,22]. The LC is a zinc-metalloprotease that cleaves one of the three SNARE proteins (SNAP-25, VAMP and syntaxin) involved in neurotransmitter exocytosis. The LC delivery across the vesicle membrane, to enter the neuronal cytosol upon acidification, is facilitated by the translocation domain located on the N-terminal domain of the H chain (HN) while the half C-terminal (HC) domain, composed of two sub-domains (HC N-terminal moiety (HCn) and HC C-terminal moiety (HCc)), is responsible for binding of the toxin to presynaptic membrane of neurons prior to endocytosis [23,24]. However, the molecular mechanism underlying membrane insertion of HN remains poorly understood.

HC binds to polysialo-gangliosides and protein receptors on neuronal membrane depending on the BoNT toxinotype [25,26], triggering the internalization by dual-receptor-mediated endocytosis followed by translocation of LC into the cytosol. The unique BoNT properties have paved the way for neuronal transport elucidation and protein-protein interaction while stimulating basic mechanistic studies [27]. Botulism occurs when BoNT has reached the peripheral nerve endings via the lymph or bloodstream subsequently to food intoxication or intestinal colonization—but mechanisms behind the absorption of BoNT through mucosal membranes have not been fully explored. The BoNT complexes containing hemagglutinin (HA) proteins disrupt the intestinal barrier by direct binding to E-cadherin and inhibition of E-cadherin-mediated cell-cell adhesion [28,29]. However, BoNT toxinotypes E and F as well as many BoNT toxinotypes A lack hemagglutinin encoding genes and are produced without hemagglutinins, yet they cause botulism. Indeed, BoNT translocation through the intestinal barrier in a mouse ligated intestinal loop model, has been shown to occur via an endocytosis-dependent mechanism while BoNT further targeted neuronal cells and extensions within the intestinal submucosa [30,31].

## 3. Current Therapeutic Anti-BoNTs Antibodies

Europe relies on a heptavalent equine anti-toxin serum (HBAT, by Cangene Corporation, Winnipeg, Manitoba, Canada and equine trivalent antitoxin serum from Behring), of which there are limited stockpiles. In addition, these equine sera bear the risk of inducing adverse effects such as serum sickness [32]. Two treatments based on passive administration of antibodies are also available in the USA but similarly with limited stock. The first one—BabyBIG^®^—which was initially based on human immunoglobulins, is supplied by the California Department of Public Health. This formulation has been recently discontinued and will be replaced with a new formulation currently under clinical development [33]. The second antidote consists of a modified HBAT (Cangene Corporation) with limitations due to the risk of serum sickness like other therapeutic equine sera [34] (Table 1). The CDC announced the availability of this new heptavalent botulinum antitoxin through a CDC-sponsored Food and Drug Administration (FDA) Investigational New Drug (IND) protocol. The HBAT replaced a licensed bivalent botulinum antitoxin AB and an investigational monovalent botulinum antitoxin E (BAT-AB and BAT-E, Sanofi Pasteur) with expiration of these products on March 12, 2010. The HBAT contains fragments of IgG targeted against seven BoNT types derived from equine plasma consisting of <2% intact IgG and ≥90% Fab or F(ab’)2 immunoglobulin (Ig) fragments to reduce the hypersensitivity reaction. The Fab and F(ab’)2 fraction being eliminated from blood circulation more rapidly than intact IgGs, makes HBAT plasma’s half-life shorter and may require additional administrations when botulism or exposure to BoNT is prolonged [35,36]. This occurred in a patient with BoNT/F intestinal botulism showing improvement after a unique HBAT administration but, when Fab/F(ab’)2 IgG fragments were cleared from the circulation, the BoNT/F rebounded and bilateral descending flaccid paralysis recurred [35].

## 4. Generation of Animal Hyperimmune Anti-BoNT Sera

Medical treatment of botulism has historically relied upon antibody therapy. Antibodies to the botulinum toxins are also used as diagnostic reagents to classify toxinotypes. Historically, formalin-detoxified toxins have been used to generate antitoxins but more recently recombinant or chemically-altered derivatives of the toxins have been proposed [37,38,39]. Improved formalin-detoxified toxoids were prepared from BoNT/A in Keller laboratory by optimizing formaldehyde reaction conditions that induced higher protective antibody titers in mice compared to commercially available formalin botulinum toxoids. This study established that the protective IgG titers induced by formalin toxoids derived from the purified BoNT protein can vary greatly depending on the reaction conditions used during the detoxification [40].

Soon after, the production of hyperimmune monovalent antitoxins to BoNT toxinotypes A and B was improved by immunizing horses with the newly developed formalin toxoids. Three horses received additional toxoid booster injections with either the Toxoid A derived from BoNT/A or Toxoid B derived from BoNT/B to induce hyperimmune antibody titers. Each monovalent plasma pool failed to cross-neutralize other BoNT toxinotypes indicating a high degree of specificity of each antitoxin for the toxinotype used during immunization [41]. In Japan, Japanese antitoxins have been used for more than 50 years; however, their safety and therapeutic efficacy are not clear. Indeed, published reports about botulism cases in which botulinum antitoxins were used, were retrospectively analyzed in terms of safety and efficacy of the therapy. A total of 134 patients administered with botulinum antitoxins were identified from published reports. Two cases of side effects (1.5%) were detected after antitoxin administration; both cases were not fatal. These data suggest that the therapy with Japanese antitoxins is safe and highly effective [42]. 

Polyclonal antibody preparations and sera used for treatment of botulism are listed in Table 1. 

## 5. Generation of Anti-BoNT Human Immunoglobulins

A recent antibody therapy, derived from the plasma of volunteers, is composed of human Igs obtained from pooled-plasma from subjects immunized with a pentavalent vaccine composed of BoNT/A, B, C, D and E toxoids. As a result, the “BabyBIG” (botulism immune globulin intravenous, human) was the antitoxin of choice for specific treatment of infant botulism in the United States [34]. However, this formulation has been recently modified due to the discontinuation of the heptavalent vaccine, which prevented the immunizations required before plasma donations. The future BIG-IV formulation will be based on a bi-valent receptor binding domain vaccine to BoNT/A and /B only. The BabyBIG antitoxin reduced hospital time for infant botulism cases by about 50% but its high cost limits its wide use. The new formulation of BabyBIG will be based on a bi-valent receptor binding domain vaccine to BoNT/A and /B [33]. 

In addition, since March 13, 2010, HBAT became the only botulinum antitoxin available in the United States for naturally occurring non-infant botulism [43]. In parallel, the effectiveness and safety of another formulation of equine botulinum antitoxin (EqBA) as an alternative treatment was assessed on cases of infant botulism registered in Mendoza, Argentina, from 1993 to 2007. Neither sequelae nor adverse effects attributable to EqBA were noticed, except for one infant who developed a transient erythematous rash. These results suggested that EqBA could be considered as an alternative specific treatment for infant botulism when BabyBIG is not available [44].

## 6. Investigations of Neutralizing Epitopes in BoNTs

Europe relies on limited stockpiles of equine antitoxin sera presenting a risk of adverse effects including serum sickness combined to short half-life and whose effectiveness against various BoNTs subtypes is unknown. To add to this complexity, vaccination of the populations is not an appropriate response to the threat due to the expanding therapeutic applications of BoNTs. The current situation highlights the urgent need for innovative and highly-effective human-like antibody preparations or inhibitors offering better tolerance than equine antibodies. In addition, the sequence variability found between BoNT toxinotypes and subtypes directly affects antibody binding and neutralization. This variability must be accounted for when generating and evaluating antibodies to neutralize specifically each toxinotype or subtype [1,32,45,46]. More importantly, optimal tolerance of antibodies is the essential criterion to be considered for human application. Since it is difficult to immunize a sufficient number of human volunteers with the antigen of interest in the case of biological agents, human volunteers may be replaced by non-human primates for the construction of immune libraries. The generation of non-human primate immune libraries combined with germline humanization techniques has led to the isolation of therapeutic antibodies close to their human counterparts, which offer improved tolerance in therapy [47,48]. 

For a better understanding of BoNT neutralization with antisera, mapping of neutralizing epitopes was developed based on experimental animal antibodies.

### 6.1. Epitope Mapping Models

**Figure 1:**
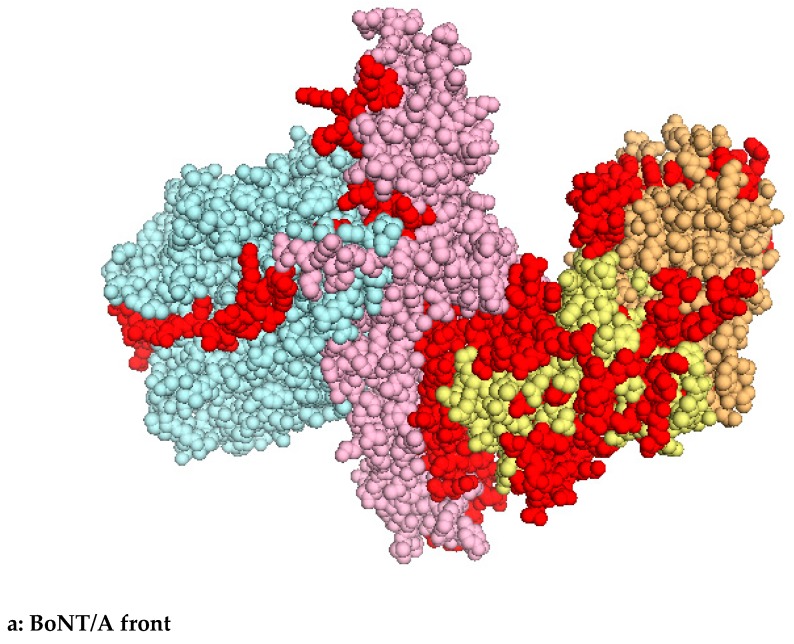

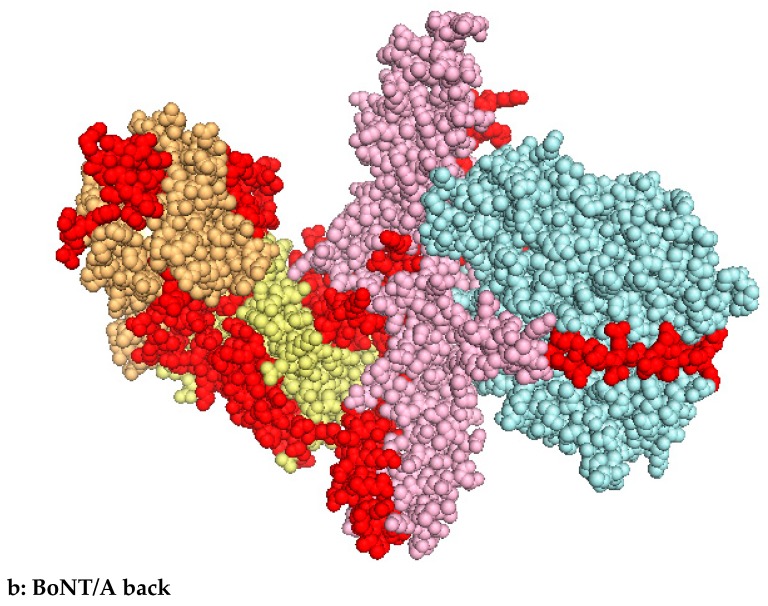
Epitope mapping of neutralizing antibodies (red); Blue: light chain (LC), pink: H chain (HN), yellow: HC N-terminal moiety (HCn) and HC C-terminal moiety (HCc).

In order to achieve better selectivity, epitope binding models were proposed by Chen and coworkers in 1997 when they illustrated the interaction between BoNT and hemagglutinins. The model was established as a guide for the design of neutralizing antibodies and to clarify how the associated non-toxic proteins protect the BoNT from proteolysis. The domain organization of BoNT/A was studied using 44 mouse monoclonal single-chain variable fragments (scFvs) selected from phage libraries. All of the covered epitopes were mapped in the binding domain of BoNT, suggesting that the binding domain is in direct contact with the nontoxic portion in the complex. Therefore, the majority of antibodies against the binding domain recognizes epitopes that must be covered when the BoNT is combined to the non-toxic complex proteins. Fifteen antibodies were deduced to bind the catalytic domain on conformational epitopes shared between domains that may not exist in the separate domains. Based on the preliminary antibody mapping to the different domains of BoNT, a model of the BoNT complex was proposed [49]. Epitope mapping of the only neutralizing antibodies is summarized in Figure 1 and Table 2 [50,51,52,53,54]. It is noteworthy that almost all the neutralizing antibodies are localized on the receptor binding domain of BoNTs and, to a lesser extent, on the translocation domain (HN). Blockade of the interaction of BoNTs with their specific receptors is an efficient mechanism of toxin neutralization. The efficacy of an individual neutralizing antibody may be hampered by the potential modification of its target epitope in a subtype of toxin. These limitations support the use combinations of several antibodies having different specificities to the target antigen. Combined antibodies preparations are better suited to improving the clearance of the agent since neutralization is more efficient when multiple antibodies bind the toxin simultaneously. Extending the half-life of antibodies used for the treatment for botulism due to food poisoning will also help to reduce the impact of prolonged toxin production in case of gut colonization by clostridial species [55]. It remains essential to distinguish the neutralization of the toxins by antibodies blocking specific functional sites such as binding to neuronal receptors or inhibiting enzymatic site from the *in vivo* clearance of the toxin from the circulation by antibodies binding to the toxin and the subsequent immune system cascades that result in renal clearance of the toxin [56]. 

### 6.2. Epitope Mapping Based on Human Antibodies

However, given that human BoNT antisera are of limited supply and equine BoNT antisera are prone to unwanted side effects, such as serum sickness and anaphylaxis, Adekar and coworkers sought to explore the human native antibody response to BoNT [57]. Native antibodies are those that arise within an intact human immune system including affinity-matured antibodies, encoded by DNA sequences that have undergone somatic hypermutation, and natural antibodies, which are encoded by un-mutated germ-line DNA sequences.

Natural antibodies that arise within an intact human immune system serve an important role in the initial antibody immune defense against viral and bacterial pathogens, creating multivalent complexes that sequester infectious agents in the spleen. Using a murine cell line and primary human B cells, they have generated two hybridomas stably secreting IgM antibodies and specifically binding to BoNT/A. Both antibodies were un-mutated IgM antibodies, consistent with an origin in naive B cells. One of the antibodies did fully neutralize a lethal dose of BoNT/A *in vivo*. They suggested this natural antibody repertoire to serve as a first-line defense against structurally non-repetitive biological toxins such as BoNT/A adding that the toxins and the natural antibody repertoire of their hosts may have been shaped by a co-evolutionary process [57]. They also applied a novel cell culture selection strategy using peripheral blood B-cells to generate libraries of stable hybridomas expressing affinity-matured, antigen-specific human IgG antibodies. This selection method followed by *in vitro* expansion in the presence of IL-4, IL-10 and CD40L reduced the background of IgM-secreting hybridomas, enriched the resultant hybridoma libraries for secretion of post-germinal center IgG antibodies, and increased the yield of antigen-specific antibodies cloned [58].

In addition to epitope mapping to different domains of BoNT, the review of the specificity of blocking antibodies produced by humans after treatment with BoNT/A or BoNT/B, and how these antibodies block the action of the correlate toxin was proposed to design peptide-based synthetic vaccines against toxin poisoning. Atassi and coworkers localized the BoNT regions that bind blocking antibodies from 28 BoNT/A- and 30 BoNT/B-treated dystonia patients who became unresponsive and whose sera protected mice against LD100 (BoNT dose causing death of 100% of the mice) of the related BoNT [59]. The continuous regions of BoNT/A LC recognized by anti-toxin antibodies from mouse, horse and chicken have also been mapped, allowing the description of the complete antigenic structure of BoNT/A. The epitopes of neutralizing antibodies on the BoNT 3-D structure show that antigenic regions are on the surface of the toxin revealing that the enzymatic activity of the LC is obstructed when the antibodies are bound [60]. Using 92 synthetic 19-residue peptides that overlapped by 5 residues and comprised an entire toxin (A or B) they determined the peptides’ ability to bind anti-toxin antibodies of human, mouse, horse and chicken. They showed that selected synthetic peptides were able to inhibit the toxin’s action *in vivo* and that epitopes designed on unresponsive patients blocking antibodies should be valuable tools in synthetic vaccines design [61].

In summary, the mapping of neutralizing epitopes using mouse monoclonal antibodies has shown that almost all the neutralizing antibodies are localized on the receptor binding domain and to a lower extent on the translocation domain (HN) of BoNTs. The blockade of the interaction of BoNTs with their specific receptors was recognized as an efficient mechanism of toxin neutralization. Moreover, the use of natural human antibodies as well as antibodies produced by unresponsive patients will be essential to achieve a better selectivity.

## 7. Generation of Mouse, Sheep or Humanized Monoclonal Antibodies 

Existing treatments against BoNTs intoxication including polyclonal antisera derived from immunized humans or horses have similar drawbacks and do not reverse paralysis completely since they act by neutralizing toxins outside the neuronal cells. Therefore, their efficacy is related to their early administration before toxin uptake by neurons. Thereby, more efforts are required to develop efficient antitoxins against BoNTs in the form of neutralizing monoclonal antibodies (mAbs) that can represent a safer and scalable therapeutic drug. MAbs provide very good prospects for enhanced neutralization potency using the synergistic effects of multivalent mixtures and improving their stability and adherence with heteropolymers or fusion proteins. Although polyclonal antisera are efficient, they are finite and suffer from substantial batch-to-batch variation. Monoclonal antibodies offer limitless amounts of a precisely targeted agent selected from an immunized animal that do not bear the risk of allergic responses or even serum sickness.

### 7.1. Benefit of mAbs Combinations or Single mAbs

Mouse mAbs against BoNT/A, B, E, and F, have been generated and characterized. Unique mAbs have also been developed to efficiently neutralize BoNT/A as well as combination of three mAbs [62,63,64,65,66,67,68,69]. However, these mAbs are specific of some BoNT/A subtypes. In a more recent study, highly specific and neutralizing mAbs against botulinum toxinotypes were generated by immunization of mice with a trivalent mixture of HC/A, B, and E in a single process. A strong synergistic effect of up to 400-fold enhancement in the neutralizing activity was obtained with toxinotype-specific mAbs combinations against BoNT/A, B, and E that may possess diagnostic and therapeutic potential [70].

Mouse mAbs against BoNT/ A, B, E, and F, were generated, which recognized their respective toxins, but also possessed neutralizing activity against BoNT/A complex *in vivo*, both individually and in mixture. The mixture of the antibodies neutralized the highest dose of the toxin. Such mAbs represent promising candidates for development of humanized therapeutic antibodies against BoNT/A-caused botulism [71]. Similarly, six highly protective sheep mAbs (SmAbs) derived from sheep immunized with BoNT/A1 toxoid or BoNT/A1 HCc have been generated. Divalent and trivalent combinations of the SmAbs, were highly protective and the trivalent combination was 100% protective against experimental clinical signs and death, reflecting protective levels not reported previously [72]. MAbs are also effective for immuno-affinity purification and concentration of BoNTs from complex matrices such as clinical samples in the mass spectrometry-based method [73].

As described above, therapeutic mAbs hold considerable promise in botulism treatment. However, neutralizing potencies of mAbs against BoNTs are usually less than that of polyclonal antibodies. As shown by Chen and coworkers, the confirmation of key epitopes is paramount to developing more efficient antibodies. By comparing the neutralizing effects among different mAbs combination groups, they found that the combination of two mAbs recognizing different receptor binding sites in BoNT/Bs had a synergistic effect [74]. More recently, the combination of 3 mAbs against BoNT/A or BoNT/B were found effective in preventing botulism after an aerosol challenge with BoNT/A1 or BoNT/B1. Those mAbs combinations represent an alternative to vaccination in those at risk of BoNT exposure [75].

Existing mAbs can also be used to build highly efficient neutralizing chimeric antibodies by selecting and shuffling VH and VL domains from a variety of repertoires. Mouse-human chimeric mAbs that could neutralize BoNTs were developed and shuffling chimeric antibodies designed with replacement of their VH or VL domains were constructed. A shuffling chimeric antibody might be the best candidate for replacing horse antitoxin to induce passive immunotherapy against botulism and avoiding the risk of serum sickness [76]. A mouse-human chimeric antibody able to neutralize BoNT/E was also developed. This chimeric antibody seems to be a useful candidate for infant botulism in which the use of passive immunotherapy with equine sera is not desirable to avoid anaphylactic shock [77]. A similar approach was used to generate a powerful chimeric antibody against BoNT/A, namely the TA12 [78]. However, while chimeric antibodies are expected to be less immunogenic, it is still possible that the rodent variable domains may remain immunogenic giving rise to an immune response. This may limit repeated long-term administration of chimeric antibodies.

Significant improvements in treatment efficacy may be obtained by using trivalent mAbs. This principle that safe and effective substitutes for polyclonal sera may be created from cloned antibodies has been demonstrated by the creation of a triplex antibody combination that is able to neutralize large doses of BoNT/A [62,64]. 

### 7.2. Optimisation of mAbs Efficiency

Following antibody treatment, toxin is mainly sequestered in the liver [79]. However, the mechanism of toxin clearance by the antibody has not been fully determined. Cheng et al. reported the *in vivo* neutralization of BoNT/A by mAbs in systemic and oral models of botulism. A window of opportunity for mAb rescue following intoxication was defined as well as antibody dosage and timing of administration. The increased knowledge in pharmacokinetics of the toxin and antibodies has allowed the establishment of a direct correlation between toxin pharmacokinetics and antibody rescue. A therapy combination based on mAbs F1-2 and F1-40 targeting both BoNT/A LC and HC significantly increased protection in a mouse intoxication model. The establishment of windows of opportunity for antibody therapeutic treatment are invaluable for future development of antibodies against intoxication by BoNT [80]. Moreover, protection from BoNT lethality and rapid BoNT clearance through the liver was evidenced in mice by administration of a pool of epitope-tagged small protein binding agents together with a single anti-tag mAb. Pharmacokinetic studies demonstrated that BoNT/A was rapidly cleared from the sera of mice given a pool of anti-BoNT/A scFvs and an anti-tag mAb but not from the sera of mice given scFvs or anti-tag mAb alone suggesting the combination of binding agents with one single anti-tag mAb as a valuable therapeutic option [56]. It was also shown that PEG treatment strongly enhanced the half-life of mAbs without affecting the effectiveness of neutralization. This prolonged mAb activity is promising for rapid treatment after BoNT/A contamination [63].

### 7.3. Benefit of Heteropolymers and Fusion Proteins

In addition to the use of trivalent anti-BoNT/A, B and E mixtures, toxin sequestration and clearance by mAbs may be improved by enhancing their ability to bind to red blood cells (RBCs). Conversion of anti-BoNT mAbs to heteropolymers (HPs) cross-linked to mAbs targeting the complement receptor, a protein that is expressed on the surface of RBCs in primates, facilitates their clearance. The HPs presented greater potency than un-modified mAbs and in a post-exposure therapeutic model, gave complete protection up to 3 h after toxin exposure. In a pre-exposure prophylaxis model, mice were fully protected from a lethal BoNT dose, demonstrating the benefit of HPs as therapeutics of BoNT intoxication [81]. It was later shown that the fusion protein complex (FP) provided the greatest benefit to the 4-mAb combination creating a 4-mAb:FP complex that dramatically enhanced potency of BoNT/A immune complexes. RBC-targeted immunoadherence is a potent enhancer of mAb-mediated BoNT/A neutralization *in vivo* and can have positive effects on BoNT/A sequestration, immune complex uptake, and macrophage activation [82]. Another method for enhancing adherence of BoNT-specific antibodies involved a fusion protein to link biotinylated molecules to glycophorin A on the RBC surface that significantly increased the potency of single and double antibody combinations. In a post-exposure model of intoxication, the complexes gave complete protection from a lethal BoNT/A1 dose when administered within 2 hours of toxin exposure. The RBC-targeted immunoadherence through FP potently enhanced the BoNT neutralization *in vivo*. [83]

## 8. Alternative Strategies for Improvement of mAbs Neutralization Efficiency

### 8.1. mAbs Targeting the LC

The majority of the efforts to generate antibodies for use as BoNT therapeutics has focused on antibodies directed against HC. The potential for an antibody directed against LC to neutralize toxin *in vitro* and *in vivo* was explored by Adekar et al. in 2008, using a novel hybridoma method for cloning human antibodies. The 4 antibodies against LC/A demonstrated potent *in vivo* neutralization when administered alone and synergized with an antibody specific of HC confirming that targeting LC may provide valuable components of an antidote against BoNT/A [84]. This was confirmed in an early study by Adekar and Takahashi when antibodies directed against LC acted at three different sites to induce neutralization. They first acted to block toxin entry into neurons, then promoted clearance of toxin from the general circulation, and finally, protected cholinergic nerves from BoNT action. These data evidenced that anti-LC antibodies may evoke three layers of protection against BoNT that form the basis of more efficient antitoxin therapies. However, since neurons were exposed to high toxin concentrations, further *in vitro* studies are needed to confirm that anti-LC antibodies are specifically taken up along with the BoNT using picomolar amount of toxin [84]. The information gained from those studies could facilitate the development of potent inhibitors that prevent the binding of BoNTs to receptors in combination with antibodies inhibiting the cleavage of SNAP25 protein. However, there is little information that defines the circumstances under which these antibodies can be used. The core observation that emerged from the work of Al-Saleem was that the window of opportunity for a post challenge administration of antibodies to exert a beneficial effect increased as the challenge dose of toxin decreased. It was suggested that the identification of factors that govern post challenge efficacy of antibodies against BoNT can be used to assess efficacy of medical countermeasures against any agent of bioterrorism or biological warfare [85].

### 8.2. Enhance Adherence and Targeting Efficiency

Another enhancing strategy employs a single recombinant “targeting agent” that binds to a toxin at two unique sites and a “clearing antibody” that binds two epitopes present on each targeting agent. Co-administration of the targeting agent and the clearing antibody resulted in decoration of the toxin with up to four antibodies to promote accelerated clearance. Surprisingly, when a post-intoxication treatment model was used, a toxin-neutralizing heterodimer agent fully protected mice from intoxication even in the absence of clearing antibody [72]. However, antibodies targeting the proteolytic domain of the toxin can inhibit the proteolytic activity of the toxin intracellularly and potentially reverse intoxication, if they can be delivered intracellularly. To generate mAbs that could reverse paralysis, the protease domain was targeted revealing that inhibitory mAbs bound near the active site, substrate-binding site or the extended substrate-binding site. The results provided mAbs that could prove useful for toxin intracellular activity reversal and relief of paralysis as well as identifying epitopes that could be targeted by small molecule inhibitors. Liposomal “delivery vehicles” could be used to ensure the delivery of antibodies into motoneurons and reverse paralysis [67,86].

To summarize, the immunogenicity potential of BoNT/A HC and LC domains were investigated extensively leading to important findings. The BoNT/A HN region has also been recently explored as a vaccine candidate indicating that the BoNT/A HN domain is a valuable region in generating protective antibodies [87]. In addition to those findings, the presence of the region connecting HCc and HCn, that has been defined as the interaction site with SV2 receptor [88], has been found essential for neutralizing antibodies generation [89].

### 8.3. Benefit of Camelid Antibodies

Three antitoxins consisting of the variable domains of camelid HC-only antibodies (VHH) were generated by expression in the chloroplast of green algae. The antibody domains bind to BoNT/A with similar affinities as camelid antibodies produced in *Escherichia coli*, and protect primary rat neurons from intoxication with BoNT/A in a similar manner. The camelid antibodies were obtained without the use of solubilization tags commonly employed in *E. coli*. These findings support the use of orally delivered antitoxins produced in green algae as a novel treatment for botulism [90]. The VHH has the unique ability to identify and bind specifically to target epitopes and allow ease of production in bacteria and yeast. The camelid VHH against BoNT/E was expressed and purified in *Pichia pastoris*. The favorable protein folding in *P. pastoris* seems to play a role in its better toxin-binding property [91]. Similarly, small (14 kDa) binding domains specific of LC/A and LC/B were selected from libraries of heavy chain only antibody domains (VHHs or nanobodies) cloned from immunized alpacas. A VHH inhibitor was able to protect neuronal cell SNAP25 protein from cleavage following intoxication with BoNT/A holotoxin demonstrating the utility of the VHH targeting domains as components of therapeutic agents against botulism intoxication [92]. However, the therapeutic use of VHHs is limited by their short half-life in the blood circulation. To overcome these limitations, mouse and human red blood cells were engineered to express VHHs against BoNT/A on their surface. Infusion of these cells into mice conferred long lasting protection against a high dose of BoNT/A [93].

### 8.4. DNA-Based Immunization

To sum up, alternative strategies may have widespread applications in antitoxin development and therapies in which neutralization and/or accelerated clearance of a toxin offer greater therapeutic benefit. However, beside the recombinant antibodies that require complex steps of production, DNA-based vaccines offer a more convenient alternative to recombinant proteins. DNA vaccination represents an effective strategy for high level production of antibodies in animals. Such antibodies can then be used as antitoxin.

The use of DNA electrotransfer into the skeletal muscle enhanced the antibody response against BoNT/A, B, and E in mice. Codon-optimized plasmid DNA encoding the highly immunogenic HC markedly increased the immune response and the corresponding neutralizing antiserum titers [94]. In parallel, to improve the yield of induced antibodies, Trott and coworkers suggested the use of an egg antibody platform for producing materials for the detection and neutralization of BoNT/A that could provide enough antibody to neutralize approximately 11.6 million mouse lethal doses (MLD) of BoNT, but this type of platform is not currently used for routine production [95].

Thanks to efficient DNA delivery techniques such as *in vivo* electroporation, it has been made possible to raise very high antibody titers in rabbits by electroporation of an antigen encoding plasmid DNA in the skeletal muscle. The neutralizing titers obtained after three treatments were high enough to fit the European Pharmacopeia for BoNT/A and /E. Furthermore, the raised antibodies have high avidity and are suitable for *in vitro* and *in vivo* immunodetection [96].

## 9. Generation of BoNT Neutralizing mAbs by Phage Display Technology

### 9.1. Construction of Immune Libraries

Phage display is a powerful technique in which peptides or proteins are expressed on the surface of bacteriophage and are selected against a target antigen. Phage display method has been proven to be a fast, cost-effective alternative for mAb generation since antibodies can be made completely *in vitro*, bypassing the immunization procedures. The selected mAbs can be easily manipulated to improve their affinity or to be converted into various antibody formats. Additionally, if human antibodies are desired, a human antibody gene repertoire as the source of phage display libraries can be directly isolated and applied to clinical trials, avoiding tedious humanization techniques [97]. In 1997, Amersdorfer and coworkers generated antibodies capable of neutralizing BoNT/A by using phage antibody libraries. Mice were immunized with BoNT/A HC, the spleens were harvested, and scFv phage antibody libraries were constructed from the Ig heavy-chain and kappa light-chain variable region genes. The results suggested the presence of two binding sites on HC which are involved in toxin internalization and toxicity [98]. In order to investigate the antibody response in botulinum intoxication, they studied the immune response to BoNT/A binding domain at the molecular level using phage display. The scFv antibodies were isolated from V-gene repertoires prepared from a human volunteer immunized with pentavalent botulinum toxoid. Epitope mapping of immune scFv binders towards BoNT/A HC surprisingly revealed a limited number of scFv recognizing conformational epitopes and clones derived from a non-immune library appeared to share no overlapping epitopes with clones derived from immunized volunteers. Therefore, they suggested that a vaccine based on the pentavalent botulinum toxoid directs the humoral immune response to a limited number of epitopes exposed on the binding domain HC [99]. It was also shown that immunizing mice with selected phage clones from naïve libraries elicited a specific humoral response against BoNT/A. These results suggested that phage-displayed random-peptide libraries are useful in the identification of neutralizing epitopes for the design of vaccines [100]. The use of a human naïve scFv phage display library generated human neutralizing mAbs against BoNT/B and F2, promising a therapeutic lead for further development [101]. By using purified BoNT/A HC fragments as antigen, two specific neutralizing antibodies mapping different epitopes were selected from a fully synthetic human antibody library. The results proved that selecting from the fully synthetic human antibody phage display library is an efficient way to obtain human therapeutic antibodies [102]. 

### 9.2. mAbs Engineering to Improve Affinity and Cross-Reactivity

Moreover, the three domains of BoNT/A can be displayed on the surface of yeast and used to epitope map six mAbs to the toxin domains they bind. Using a library of yeast-displayed BoNT/A HC mutants, the epitopes of three neutralizing BoNT/A mAbs were identified. Epitopes modeling showed all three mAbs binding to BoNT/A simultaneously and may explain, in part, the dramatic synergy observed on *in vivo* toxin neutralization when these antibodies are combined [50]. Yeast mating was used to affinity mature BoNT antibodies by shuffling the wild-type light chain variable region gene of the Ig. This technique provides a rapid route to antibody affinity maturation. A library of Ig light chains was generated in a yeast vector where the light chain is secreted. The heavy chain variable region and the first domain of the constant region from a mAb was cloned into a different yeast vector for surface display as a fusion protein. The affinities of one BoNT/A and two BoNT/B scFv antibody fragments were increased from 9- to more than 77-fold by yeast mating [103]. Yeast-displayed scFv antibodies generated from vaccinated humans or mice were tested for their ability to bind multiple BoNT toxinotypes. Using molecular evolution techniques, it proved possible to both increase affinity and maintain cross-toxinotype reactivity for the antibodies that potently neutralized BoNT *in vivo* [68].

In addition to human libraries, dromedary derived libraries can generate efficient antibodies repertoires. A single-domain VHH fragment was obtained from an immune dromedary phage display library against the putative binding domain of BoNT/E. The characteristics of nanobody VHH include excellent production, superior heat stability and specific binding capacity to soluble antigen without cross-reaction to other relevant or irrelevant antigens confirming the unique properties of the nanobody applicable in diagnostics or therapeutic purposes [104].

ScFv libraries from immunized humans and mice were displayed on the surface of yeast. The fine epitopes of selected mAbs were mapped by alanine-scanning mutagenesis, revealing that the inhibitory mAbs bound one remote region from the BoNT/A LC catalytic center. The results provide mAbs that could prove useful for intracellular reversal of paralysis post-intoxication and further define epitopes that could be targeted by small molecule inhibitors [67].

The botulinum strain IBCA10-7060 produces the recently described BoNT/FA or HA hence, more potent and safer antitoxins against BoNT/FA or HA are needed. The existing mAbs to BoNT/A and BoNT/F were evaluated for BoNT/FA or HA binding and yeast-displayed mutants created to select higher-affinity-binding mAbs by using flow cytometry. This 3-mAbs combination potently neutralized BoNT/FA or HA and represents a potential human antitoxin that could be developed for the prevention and treatment of type FA or HA botulism [105]. The toxicity in animals was completely neutralized using a combination of serotype B and A antitoxins while no other combination of antitoxins protected the animals. In addition, BAT (heptavalent antitoxin) eliminated the toxic effects of both BoNTs, suggesting that current therapeutic treatment products would likely be effective in individuals exposed to this hybrid toxin [14]]. Subsequent studies using fully active BoNT/FA showed significant neutralization with HBAT [106].

A co-formulation of an antitoxin to the three most important toxinotypes is currently developed. The mAbs were generated from humans immunized with pentavalent toxoid via phage or yeast antibody libraries. The combination of these antibodies obviates the need to identify the toxinotype causing intoxication to prevent botulism resulting from exposure to BoNT/A, B, or E. This work confirmed the feasibility of oligoclonal antibody therapies for biodefense that would facilitate stockpiling, distribution, and administration [107]. In addition, a derivative of three-antibody combination (NTM-1633) is in pre-clinical development for neutralization of BoNT/E1, BoNT/E3, and BoNT/E4. Libraries of yeast-displayed scFv antibodies were created from variable region genes of humans immunized with pentavalent-toxoid- and the BoNT/E-binding scFv isolated by Fluorescence-Activated Cell Sorting (FACS) [106]. An equimolar combination of the three mAbs was able to potently neutralize BoNT in a mouse neutralization assay [69].

As current antitoxin therapies are scarce and produce adverse reactions, the XOMA3AB was developed in the United States based on a human phage library, as a potential therapy for the treatment of BoNT/A disease. XOMA 3AB is an equimolar mixture of three IgGs, that target different BoNT/A regions and have been engineered to bind BoNT/A1, A2, A3, and A4. Safety findings support further investigation of XOMA 3AB as a potential agent for botulism treatment and post-exposure prophylaxis [108]. 

However, due to very limited availability of anti-BoNT stocks outside USA, there is a pressing need to stockpile similar countermeasures in Europe. The aim of the AntiBotABE Program was the isolation of recombinant antibodies neutralizing BoNT/A, B and E. Starting from the generation and panning of 6 immune phage-display libraries of macaque origin (*Macaca fascicularis*) a selection of recombinant antibodies neutralizing BoNT/A, B and E were isolated using *ex vivo* and *in vivo* protection models. Six macaques were immunized with the recombinant and non-toxic HC and LC of BoNT/A, B and E. The framework regions of macaque antibodies were made more similar to the corresponding human germline sequences by calculating the level of identity of the corresponding regions to the most similar human germline-encoding framework regions. For each library, the best *ex vivo* neutralizing antibody fragments were germline-humanized and expressed as IgGs. The selected IgGs were tested in a standardized *in vivo* mouse protection assay and challenged with BoNTs produced from collections of *Clostridium* strains. Interestingly, protective antibody combinations against BoNT/A and BoNT/B were evidenced that cross-neutralized both BoNT/A1 and A2 and BoNT/B1 and B2, and for BoNT/E, the anti-LC antibody alone was found highly protective. The germline-humanized IgG hu8ELC18 demonstrated protection and prophylaxis capacity against BoNT/E in a mouse model. In addition, hu8ELC18 protected mice from lethality when injected up to 14 days prior to intraperitoneal administration of 5MLD BoNT/E. Cross-neutralizing antibodies are highly sought-after in therapy to expand the therapeutic spectrum of each single antibody while decreasing the number of antibodies required in a wide spectrum mixture [46].

## 10. Vaccination Against Botulism

BoNTs are structurally related to tetanus toxin (TeNT) that are responsible for tetanus. The vaccination based on tetanus toxoid is an efficient and universal strategy for tetanus prevention. Similarly, botulism can be efficiently prevented with BoNT toxoid. However, BoNTs are unique toxins of their kind, having both toxic effects and therapeutic benefits, thus excluding vaccination as a widely applied measure. Vaccines against botulism have been developed but vaccination is scarcely used since its effectiveness has not been fully evaluated; botulism is a rare disease and more importantly, vaccination could impact therapeutic applications of BoNTs and its safety is questionable [36]. Thus, vaccination is restricted to individuals with a high risk of exposure to BoNTs such as health care providers, researchers, first responders, and military personnel [109]. However, CDC has discontinued the botulinum vaccine program for workers at risk for occupational exposure of BoNT since 2011 due to low efficiency [32]. Indeed, BoNTs have been introduced as a safe and effective treatment for a wide range of medical disorders preventing large scale vaccination against botulism ([110,111,112]). Minute doses of this deadly poison are increasingly used therapeutically to locally paralyze muscles for clinical or cosmetic benefit. Initially used to treat strabismus, BoNT has now more than a hundred possible medical applications including movement disorders, hemifacial spasm, essential tremor, tics, writer’s cramp, cervical dystonia, cerebral palsy, hyperhydrosis and vascular cerebral stroke. Moreover, BoNT has been approved for chronic pain, migraine headache, overactive bladder and inflammation [113,114,115]. Clinical studies are currently describing its potential application in major depressive disorders [116,117,118].

## 11. Future BoNT Vaccines and Neutralizing Antibodies Development

Human mAbs may be beneficial in prophylaxis since their half-life can be extended by several techniques such as the PEG treatment or the combinations. Equine antitoxin has a shorter half-life causing potential relapse of botulism while a cocktail of mAbs is able to induce a rapid clearance of the toxin via the liver. Moreover, the level of protection conferred by mAbs is higher since quantities injected exceed that elicited by vaccines injection. However, since already intoxicated neurons cannot be rescued, antibody treatment only prevents further exposure of the toxin whatever the antibody structure. To overcome this limitation, the vaccination is essential for the population at risk of exposure such as military or laboratory personnel. [119].

To avoid undesirable side effects, vaccines against botulism have been focused on virus-vectored recombinant vaccines or recombinant nontoxic BoNT subunits. The crystallographic structures of BoNTs allow the design of specific gene sequences that can be expressed by vector systems. Those results together with the scalability of the *in vitro* protein expression systems offer alternative routes for the preparation of vaccines. Several viral-based platforms have been developed for expression of immunizing doses of BoNT based on HC and in various models. Plasmids containing genes encoding for BoNT-HCs are attractive vaccine platforms since they offer the opportunity to produce large and nontoxic quantities of BoNT domains or subunits [94]. Replicon DNA or particle pentavalent vaccines could simultaneously induce antibody responses and protect against the 5 agents including BoNT/A, B, E, F and tetanus neurotoxin. Moreover, the reverse genetic approaches generate chimeric protein that can be effectively incorporated into virions to induce antibody response [36,120,121,122,123]. Yet, the DNA vaccination in humans still presents some concerns. DNA vaccines do not contain an actual infectious agent, whether dead or alive as opposed to the classic methods of vaccination. The risk of reversion of the vaccine are not relevant to DNA vaccines, which could potentially be given to anyone regardless of health status. DNA vaccines can also stimulate the immune response, providing a person with long lasting immunity after a small number of doses. However, there are still obstacles limiting the uses of DNA vaccines, since this type of vaccination would be limited to pathogens with a distinctive protein immunogen. DNA vaccines also present a slight risk of potentially disrupting normal cellular processes. The basis for efficacy of the recombinant botulinum vaccine against toxinotypes A and B is that neutralizing antibodies bind to BoNT complex of toxinotypes BoNT/A1and BoNT/B1 and prevent their actions at cholinergic neurons. It was suggested to verify the protective capacity of human neutralizing antibodies induced by vaccination using a guinea pig passive immunization model. Estimate of the neutralizing efficiency was established for BoNT/A1 and BoNT/B1 neutralizing antibodies obtained from clinical volunteers vaccinated with recombinant botulinum vaccine A/B [124]. The recombinant vaccine based on HC/A and HC/B made on *P. pastoris* has been approved by FDA [109].

Mice were also successfully protected from 10 MLD50 BoNT/A1 when a recombinant, replication-incompetent, adenoviral vector (Ad/VNA-BoNTA) that induces secretion of biologically active VHH-based neutralizing agents (VNAs) was administered up to 1.5 hours post-intoxication. The genetic delivery of VNAs promises to be an effective method of providing prophylactic protection by demonstrating rapid appearance of the protective VNA in serum following treatment [72]. A trivalent cocktail synthetic DNA vaccine induced robust humoral and poly functional CD4C T-cell responses which fully protected animals against lethal challenge with BoNT/A, B, and E after just 2 immunizations. These data demonstrated the protective efficacy induced by a combinative synthetic DNA vaccine and the importance of the development of multivalent vaccines that provide protective immunity against a range of BoNTs [125]. Numerous human adenoviruses toxinotypes, and a replication-incompetent human toxinotype 5 (AdHu5) have been developed as a viral vaccine vector. Those viral vaccines protected mice from a challenge with 100 MLD50 of BoNT/A at seven weeks post-vaccination [126].

However, although neutralizing epitopes have been well established within the HC, the vaccination with HC subunits are often suboptimal requiring multiple immunization to achieve full protection [127]. Targeting the antigens to dendritic cells and granulocyte-macrophage colony-stimulating factor has been shown to increase vaccine efficacy. Various approaches have been implemented to further improve vaccines efficiency such as the sublingual immunization with BoNT/A HC fused to adenovirus-protein that conferred significant protective immunity [128]. The optimization of vaccines efficacy is a key factor in providing active immunity against BoNTs to at-risk population, but also essential to obtain efficient Igs based passive immunization [41,45].

Botulinum toxoid vaccines have been produced and used in Japan. However, since clinical studies involving these vaccines were conducted before establishment of the Ethical Guidelines for Clinical Research in Japan, their immunogenicity and safety were not systematically assessed. A new tetravalent (type A, B, E, and F) botulinum toxoid vaccine was assessed through quality control tests with reference to the Minimum Requirements in Japan for adsorbed tetanus toxoid vaccine. Neutralizing antibody titers for each type of toxin in the participant’s sera, 1 month after the 4th injection indicated sufficient protection. This study demonstrated that the vaccine has marked immunogenicity and is safe for use in humans [129].

Smith, and their team from USAMRIID, reported on the production of recombinant BoNT toxin domain subunits as vaccine candidates against multiple serotypes. This ciBoNT HP (catalytically inactive holotoxin vaccines) vaccine elicited a more robust neutralizing antibody response providing better protection against a challenge with the parental toxins or with dissimilar subtypes [130]. In addition, a recombinant host cell receptor-binding subunit designed for use as a potential vaccine candidate against BoNT/A has recently completed phase 2 clinical trials. The vaccine candidate was the BoNT/A HC with a point mutation BA15 (R1269A) within the ganglioside-binding site. The inability of BA15 to bind ganglioside shows that BA15 is a potential safe vaccine candidate [131]. In order to replace the current BabyBIG formulation, the future BabyBIG will be based on a bi-valent receptor binding domain vaccine to BoNT/A and /B. To ensure a continuing supply of BabyBIG, the safety and immunogenicity of rBV A/B was demonstrated in a recent Phase 2b clinical study [33].

A more recent study evidenced a dendritic cells (DC)-targeted DNA vaccine inducing stronger HC-specific humoral immune responses, lymphocyte proliferative responses and protective potency against BoNT/A in mice, showing that a DC-targeted fusion DNA vaccine could generate strong immunity against BoNTs or other pathogens in an animal model [132]. A recombinant, full-length BoNT/A1 was detoxified by engineering 3-amino acid mutations into the LC to eliminate catalytic activity, and one additional mutation in the ganglioside binding pocket, resulting in reduced receptor binding. Overall, the engineered full length BoNT/A with defects in multiple toxin functions, elicited a potent immune response to BoNT/A challenge, validating this strategy against botulism and other toxin-mediated diseases [119]. 

## 12. Concluding Remarks and Perspectives

The current status of the antitoxins and vaccines against BoNTs show the multiple promising strategies available in the treatment and prevention of botulism. A particular focus was made on strategies involving the phage display techniques for the development of novel and better tolerated antibodies. We also outlined the major steps involved in the generation of new vaccines and their current challenges. To sum up the current status, previous efforts have resulted in less than satisfactory countermeasures due to a poor tolerability, limited supply and batch to batch variation of polyclonal antibodies. Significant progress has been made using mAbs development, but further progress is still required to address the BoNTs great variability as well as the emergence of BoNT sequences in non-clostridial strains.

## Figures and Tables

**Table 1:** Polyclonal antibody preparations and sera used for treatment of botulism.

Preparation Name	BoNT Type Neutralized	Company	Antitoxin Titer	Recommended Dose
BAT-AB and BAT-E	A, B and E	Sanofi Pasteur	BoNT Type/Neutralizing Units/mL	Expired in 2010
Heptavalent equine anti-toxin (HBAT)	A–G	Cangene Corporation (USA)	BoNT/A 4500 BoNT/B 3300 BoNT/C 3000 BoNT/D 600 BoNT/E 5100 BoNT/F 3000 BoNT/G 600	1 vial (adult > 17 years) 20–100% of adult dose for pediatric (1–17 years) 10% of adult dose for infants (<1 year)
Trivalent equine anti-toxin	A, B, E	Behring (Germany)	BoNT/A 187500 BoNT/B 125000 BoNT/E 12500	2 bottles
Trivalent equine anti-toxin	A, B, E	Biomed (Poland)	BoNT/A 5000 BoNT/B 5000 BoNT/E 1000	1–5 vials
Trivalent equine anti-toxin	A, B, E	Instituto Butantan (Brazil)	BoNT/A 7500 BoNT/B 5500 BoNT/E 5000	1 vial
Bivalent equine anti-toxin EqBA	A, B	Argentina Public Department of Health	BoNT/A 7500 BoNT/B 5500	1 vial
Tetravalent equine anti-toxin	A, B, E, F	Chiba Serum (Japan)	BoNT/A 10000 BoNT/B 10000 BoNT/E 10000 BoNT/F 4000	1–3 vials
Human botulism immune globulin (Baby-BIG)^†^	A, B	California Department of Public Health (USA)	BoNT A 15 BoNT/B 4	Infant (<1 year) 1 ml/kg of body weight

^†^: BabyBIG is no longer produced as described in the text, the future BabyBIG formulation will be based on a bi-valent receptor binding domain vaccine to BoNT/A and /B only [33]. BoNT: botulinum neurotoxin,

**Table 2:** BoNT/A1 amino acid determination of neutralizing epitopes shown in Figure 1.

Binding Domain		Reference
HC domain:	C25: 889 to 1294	Mullaney et al. 2001 Levy et al. 2007
	HuC25: 918–920; 953; 1061–1066	
	3D12: 1127–1131; 1131–1264	
	S25: 1115–1223; 1254–1256	
HN domain:	N5: 505–523	Atassi 2005
	N6: 519–537	
	N8: 547–565	
	N25: 785–803	
	N26: 799–817	
Hc domain:	C2: 869–887	
	C3: 883–901	
	C4: 897–915	
	C9–11: 967–1013	
	C13: 1023–1041	
	C15: 1051–1069	
	C23: 1163–1181	
	C25: 1191–1209	
	C31: 1275–1296	
	N28: 827–845	
HN domain:	455–662	Dertzbaugh 1996
HC domain:	1150–1289	
